# DESIGNING AND IMPLEMENTING A RESIDENT-TO-RESIDENT AGGRESSION PREVALENCE STUDY IN A NEW CONTEXT

**DOI:** 10.1093/geroni/igad104.0149

**Published:** 2023-12-21

**Authors:** David Burnes, Vasuki Shanmuganathan, Pamela Baxter, Sandra Hirst, Adriana Shnall, Lynn McDonald

**Affiliations:** University of Toronto, Toronto, Ontario, Canada; University of Toronto, Toronto, Ontario, Canada; McMaster University, School of Nursing, Hamilton, Ontario, Canada; University of Calgary, Calgary, Alberta, Canada; Baycrest, Toronto, Ontario, Canada; University of Toronto, Toronto, Ontario, Canada

## Abstract

Resident-to-resident aggression (RRA) is a prevalent issue in long-term care (LTC) settings with serious consequences. Research from the U.S. has found that approximately 20% of residents experience RRA each month and this issue is associated with several physical/mental health morbidities. Very little RRA research has been conducted in LTC settings in Canada. This study sought to estimate the prevalence of RRA in Canadian nursing homes and identify risk factors from different levels of social-ecological influence. Specifically, this study sought to replicate a gold standard RRA prevalence study in the U.S. by selecting a random sample of nursing homes (n = 10) across the province of Ontario and collecting RRA prevalence data using multiple sources/methods, including staff interviews, resident interviews, and direct observation from staff and research assistants. As this study is in early stages of design/implementation, the current presentation will focus on various issues requiring consideration and the challenges faced when setting up a large-scale RRA prevalence study. Specifically, the presentation will focus on the adaptation of existing measurement tools to a new context with unique local language, legal and legislative frameworks, and the development of a sampling strategy requiring considerations related to sample inclusion/exclusion criteria, temporal and geographical logistical concerns, and sample size. We will also discuss new realities and heightened sensitivities involved in conducting LTC-based research and recruiting nursing homes, residents and staff during the COVID era that includes staffing instabilities/shortages and outbreaks. This presentation will assist other researchers around the world in planning/implementing RRA prevalence study designs.

